# Efficacy hierarchy and absolute survival benefit of first-line immunotherapy in advanced gastric cancer: a meta-analysis and pooled survival analysis

**DOI:** 10.3389/fimmu.2026.1866848

**Published:** 2026-08-17

**Authors:** Jialun Lv, Krishnapriya Thangaretnam, Md Obaidul Islam, Jason Tang, Sabine Denize, Nadeem Bhat, Shoumin Zhu, Heng Lu, Dunfa Peng, Wael El-Rifai, Zheng Chen

**Affiliations:** 1 Department of Surgery, Miller School of Medicine, University of Miami, Miami, FL, United States; 2 Sylvester Comprehensive Cancer Center, University of Miami, Miami, FL, United States; 3 American Heritage Schools, Broward Campus, Plantation, Florida, FL, United States; 4 Doctors Charter School of Miami Shores, Miami shores, FL, United States; 5 Department of Veterans Affairs, Miami Healthcare System, Miami, FL, United States

**Keywords:** chemotherapy, gastric cancer, gastroesophageal junction, immunotherapy, PD-1 inhibitor

## Abstract

**Background:**

While immune checkpoint inhibitors (ICIs) have revolutionized first-line treatment for advanced gastric and gastroesophageal junction (GEJ) adenocarcinoma, outcomes across phase III trials remained heterogeneous. This study aimed to determine the efficacy and safety of ICI plus chemotherapy, and to define a clinically meaningful PD-L1 expression threshold through a meta-analysis.

**Methods:**

We systematically searched PubMed, Embase, and Cochrane for phase III randomized controlled trials (up to November 2025) comparing ICI-chemotherapy combinations against chemotherapy alone in patients with advanced gastric or GEJ adenocarcinoma. The combined hazard ratios (HRs) for overall survival (OS) and progression-free survival (PFS) were calculated using a random-effects model. Safety was assessed through the combined risk ratio (RR) of grade ≥3 adverse events (AEs) and serious adverse events (SAEs).

**Results:**

Eight trials involving 7,127 patients with previously untreated, HER2-negative, advanced gastric or GEJ adenocarcinoma were included. The pooled analysis showed a significant improvement in OS (HR = 0.79) and PFS (HR = 0.71) with immunochemotherapy versus chemotherapy alone. Immunochemotherapy was associated with longer median OS (14.4 vs 12.6 months) and PFS (7.3 vs 6.2 months). Treatment benefit increased with higher PD-L1 combined positive score (CPS): OS HRs were 0.90 (CPS < 1), 0.76 (CPS ≥ 1), 0.74 (CPS ≥ 5), and 0.65 (CPS ≥ 10). Combination therapy modestly increased grade ≥3 AEs (RR = 1.16) and SAEs (RR = 1.54) compared with chemotherapy alone.

**Conclusions:**

Our results supported the strategy of first-line immunochemotherapy, which significantly prolongs OS and PFS in advanced gastric and GEJ adenocarcinoma with manageable toxicity.

**Systematic review registration:**

https://www.crd.york.ac.uk/PROSPERO/view/CRD420251180445, identifier CRD420251180445.

## Introduction

Gastric and gastroesophageal junction (GEJ) adenocarcinomas impose a substantial global burden, ranking fifth in incidence and fourth in cancer mortality worldwide (1). Despite advances in surgery and systemic therapy, outcomes in advanced settings remain poor. Median overall survival (OS) with fluoropyrimidine–platinum doublets rarely exceeds 12 months (2). Hence, developing systemic strategies that deliver meaningful survival gains has become a central aim in contemporary gastric cancer research.

Immune checkpoint inhibitors (ICIs) directed against programmed cell death-1 (PD-1) or its ligand PD-L1 have fundamentally changed the treatment landscape for several solid tumors, including gastric and GEJ adenocarcinoma (3). Introducing ICIs into first-line therapy rests on a biological advantage: cytotoxic chemotherapy may increase tumor antigen release and presentation, enhance T-cell infiltration, and mitigate tumor-driven immunosuppression, thereby augmenting PD-1 blockade (4). Multiple large phase III randomized controlled trials (RCTs), including CheckMate-649, ATTRACTION-4, KEYNOTE-062, ORIENT-16, and RATIONALE-305—have tested PD-1 inhibitors such as nivolumab, pembrolizumab, sintilimab, and tislelizumab in combination with chemotherapy for advanced patients (3, 5–8).

The findings of these pivotal trials have been inconsistent. CheckMate-649 showed clinically meaningful improvements in OS and progression-free survival (PFS), particularly in tumors with a PD-L1 combined positive score (CPS) ≥5, whereas ATTRACTION-4 improved PFS without an OS advantage in an all-Asian cohort (5, 6). KEYNOTE-062 reported mixed results, with limited benefit for pembrolizumab plus chemotherapy over chemotherapy alone in patients with PD-L1 CPS ≥1 tumor (9). These discrepancies may stem from variations in PD-L1 testing assays, CPS thresholds, chemotherapy backbones, or ethnic composition of the enrolled patients (10).

However, the predictive role of PD-L1 remains unsettled. Although CPS is widely used, the optimal clinical cut-off has not been firmly defined (11). Emerging subgroup analyses also suggest that age, sex, Eastern Cooperative Oncology Group performance status (ECOG PS), liver metastases, and tumor location may influence treatment effects (12, 13). A rigorous synthesis of phase III data is therefore needed to quantify the magnitude and consistency of benefits from ICI-based regimens and to identify patient groups most likely to derive a survival advantage.

Consequently, we conducted a systematic review and meta-analysis of phase III RCTs comparing first-line ICI plus chemotherapy versus chemotherapy alone in advanced gastric and GEJ adenocarcinoma. The primary objectives were to: (1) estimate pooled effects on OS and PFS; (2) examine effect modification by PD-L1 CPS and key clinicopathologic subgroups; and (3) characterize the safety profile of ICI-based combinations.

## Methods

### Search strategy and selection criteria

This systematic review and meta-analysis were conducted in accordance with the Preferred Reporting Items for Systematic Reviews and Meta-Analyses (PRISMA) guidelines (14). The research protocol was registered on the International Prospective Register of Systematic Reviews (CRD420251180445). A comprehensive search of PubMed, Embase, and the Cochrane Central Register of Controlled Trials (CENTRAL) was performed up to November 2025, using the following keywords and Medical Subject Headings (MeSH) terms:

*(“gastric cancer” OR “gastroesophageal junction cancer” OR “stomach neoplasms”)* AND *(“immune checkpoint inhibitor” OR “PD-1” OR “PD-L1” OR “nivolumab” OR “pembrolizumab” OR “sintilimab” OR “tislelizumab” OR “cadonilimab” OR “sugemalimab”)* AND *“randomized controlled trial”*.

Only phase III randomized controlled trials (RCTs) comparing ICI plus chemotherapy versus chemotherapy alone as the first-line treatment for advanced or metastatic gastric or GEJ adenocarcinoma were included.

### Inclusion and exclusion criteria

Eligible studies met the following criteria: (1) randomized phase III design; (2) patients with histologically confirmed, unresectable or metastatic gastric or GEJ adenocarcinoma; (3) Intervention: PD-1 or PD-L1 inhibitor in combination with platinum–fluoropyrimidine chemotherapy; (4) comparator: chemotherapy alone; (5) reported hazard ratios (HRs) and 95% confidence intervals (CIs) for OS or PFS.

The exclusion criteria were as follows: (1) phase I/II trials, (2) neoadjuvant or adjuvant settings, (3) single-arm studies, or (4) duplicated datasets.

### Data extraction and quality assessment

Two reviewers independently abstracted the trial-level characteristics using a standardized extraction form: study design, sample size, follow-up duration, PD-L1 testing platform, and HRs for OS and PFS. Where reported, we also captured subgroup HRs stratified by CPS threshold, age, sex, Eastern Cooperative Oncology Group performance status, liver metastases, tumor location, and geographic region. Disagreements were reconciled through discussion.

The methodological quality of each trial was appraised using the Cochrane Risk of Bias 2.0 tool (15), covering random sequence generation, allocation concealment, blinding, completeness of outcome data, and selective outcome reporting.

### Statistical analysis

We pooled HRs and 95% CIs for OS and PFS using the DerSimonian–Laird random-effects model to accommodate between-trial heterogeneity. For binary safety outcomes—grade ≥3 adverse events (AEs) and serious adverse events (SAEs)—risk ratios (RRs) were synthesized via the Mantel–Haenszel random-effects approach.

The DerSimonian–Laird random-effects model was selected as the primary pooling method because it provides conservative variance estimates and facilitates comparisons with prior meta-analyses in gastric cancer. Although restricted maximum-likelihood (REML) estimation can offer less biased between-study variance estimates, particularly with a large number of trials, simulation studies suggest that the DerSimonian–Laird method performs comparably when the number of included studies is small (n < 10). Given that this meta-analysis incorporated eight phase III trials, the DerSimonian–Laird approach was considered statistically appropriate and ensured methodological consistency with previously published immunotherapy meta-analyses.

Heterogeneity was quantified using Cochran’s Q test (significance threshold *P* < 0.10) and the *I*^2^ statistic, interpreted as low (25%), moderate (50%), or high (≥75%). When ten or more studies were available, we examined small-study effects and potential publication bias through Egger’s regression and funnel plots.

Prespecified subgroup analyses examined effect modification by PD-L1 CPS (<1, ≥1, ≥5, ≥10), age (<65 versus ≥65 years), sex, ECOG PS (0 versus 1), presence of liver metastases, tumor location (gastric versus GEJ), and geographic region (Asia versus non-Asia). Sensitivity analyses sequentially omitted individual trials to test the robustness of the summary estimates.

We reconstructed individual patient data (IPD) from published Kaplan–Meier curves using a two-stage algorithmic framework described by Guyot et al., implemented through the R package *IPDfromKM (*16). In the first stage, we digitized each curve and extracted the time points and the corresponding survival probabilities for each treatment arm. In the second stage, we integrated the extracted coordinates with reported numbers at risk and total sample sizes at each specified time point to estimate event and censoring patterns, thereby generating patient-level time-to-event data. The reconstructed IPD from all eligible trials were pooled into a unified analysis dataset. Reconstruction accuracy was assessed by comparing the reproduced risk tables, hazard ratios, and overall shapes of the regenerated Kaplan–Meier curves with the values reported in the original publications.

All computations were performed in R (version 4.5.0) using the meta and meta for packages. Forest, funnel, and Galbraith radial plots were generated using custom scripts built on a meta-framework.

### Network meta-analysis

To ascertain the comparative efficacy of various immune checkpoint inhibitors, a network meta-analysis was formulated within a frequentist framework utilizing the *netmeta* package in R. The network geometry was primarily star-shaped, predicated on chemotherapy as the common comparator node. Treatment hierarchies were quantitatively evaluated employing P-scores, a frequentist analog to the surface under the cumulative ranking curve (SUCRA). P-scores measure the extent of certainty that a specific intervention encompasses superior efficacy relative to competing regimens, bounded between 0 and 1.

### Outcomes

The primary outcome was OS. Secondary outcomes included PFS, subgroup efficacy across prespecified clinical and biomarker strata, and treatment-related toxicity (grade ≥3 AEs and SAEs).

## Results

### Study selection and characteristics of included studies

A total of 161 records were identified through the initial database search, of which eight phase III randomized controlled trials met the inclusion criteria and were included in the final analysis (5–9, 17–19) (**Supplementary Figure 1**). These trials collectively enrolled 7,127 patients with previously untreated, HER2-negative, advanced or metastatic gastric or GEJ adenocarcinoma.

All trials compared PD-1 inhibitor plus platinum-fluoropyrimidine chemotherapy with chemotherapy alone. The ICIs included nivolumab (CheckMate-649; ATTRACTION-4), pembrolizumab (KEYNOTE-062; KEYNOTE-859), sintilimab (ORIENT-16), tislelizumab (RATIONALE-305), cadonilimab (COMPASSION-15), and sugemalimab (GEMSTONE-303). All studies were double-blind, except for CheckMate-649, an open-label study.

Baseline patient characteristics were well balanced across treatment arms, with median ages ranging from 60 to 63 years and approximately 72% of patients being male. The proportion of participants with a PD-L1 CPS ≥1 ranged from 40% to 85%. Individual study details, including the trial design, population, and reported HRs for OS and PFS, are summarized in **Table 1**.

**Table 1:** Characteristics of the included studies.

Study	Year	Design	Intention-to-treat population	Experimental arm	Control arm	Population characteristics	OS (95% CI)	PFS (95% CI)
ATTRACTION-4	2022	Phase 2-3, double-blind RCT	ICI+CT: 362 CT: 362	Nivolumab plus CT	Placebo plus CT	HER2 negative, untreated, unresectable advanced or recurrent G/GEJ adenocarcinoma.	0.90 (0.75-1.08)	0.68 (0.51-0.90)
CHECKMATE 649	2021	Phase 3, open label RCT	ICI+CT: 789 CT: 792	Nivolumab plus CT	CT	Untreated, unresectable, non-HER2-positive G/GEJ cancer, and esophageal adenocarcinoma.	0.80 (0.68-0.94)	0.77 (0.68-0.87)
COMPASSION- 15	2025	Phase 3, double-blind RCT	ICI+CT: 305 CT: 305	Cadonilimab plus CT	Placebo plus CT	Untreated, HER2 negative unresectable locally advanced or metastatic G/GEJ adenocarcinoma.	0.62 (0.50-0.78)	0.53 (0.44-0.65)
GEMSTONE- 303	2025	Phase 3, double-blind RCT	ICI+CT:241 CT: 248	Sugemalimab plus CT	Placebo plus CT	Untreated, no known HER2-positive status, PD-L1 expression ≥ 5%, unresectable advanced or metastatic G/GEJ adenocarcinoma.	0.75 (0.61-0.92)	0.66 (0.54-0.81)
KEYNOTE-062	2020	Phase 3, double-blind RCT	ICI+CT: 257 CT: 250	Pembrolizumab plus CT	Placebo plus CT	PD-L1 CPS ≥ 1, HER2 negative, untreated, locally advanced, unresectable, or metastatic G/GEJ adenocarcinoma.	0.85 (0.70-1.03)	0.84 (0.70-1.02)
KEYNOTE-859	2023	Phase 3, double-blind RCT	ICI+CT: 790 CT: 789	Pembrolizumab plus CT	Placebo plus CT	Untreated, locally advanced or metastatic HER2 negative G/GEJ adenocarcinoma.	0.78 (0.70-0.87)	0.76 (0.67-0.85)
ORIENT-16	2023	Phase 3, double-blind RCT	ICI+CT: 327 CT: 323	Sintilimab plus CT	Placebo plus CT	Unresectable, no known HER2 positive status, locally advanced or metastatic G/GEJ adenocarcinoma.	0.77 (0.63-0.94)	0.64 (0.52-0.77)
RATIONALE-305	2024	Phase 3, double-blind RCT	ICI+CT: 501 CT: 496	Tislelizumab plus CT	Placebo plus CT	HER2 negative locally advanced unresectable or metastatic G/GEJ adenocarcinoma.	0.80 (0.70-0.92)	0.78 (0.67-0.90)

### Efficacy in intention-to-treat (ITT) population

**Figure 1:**
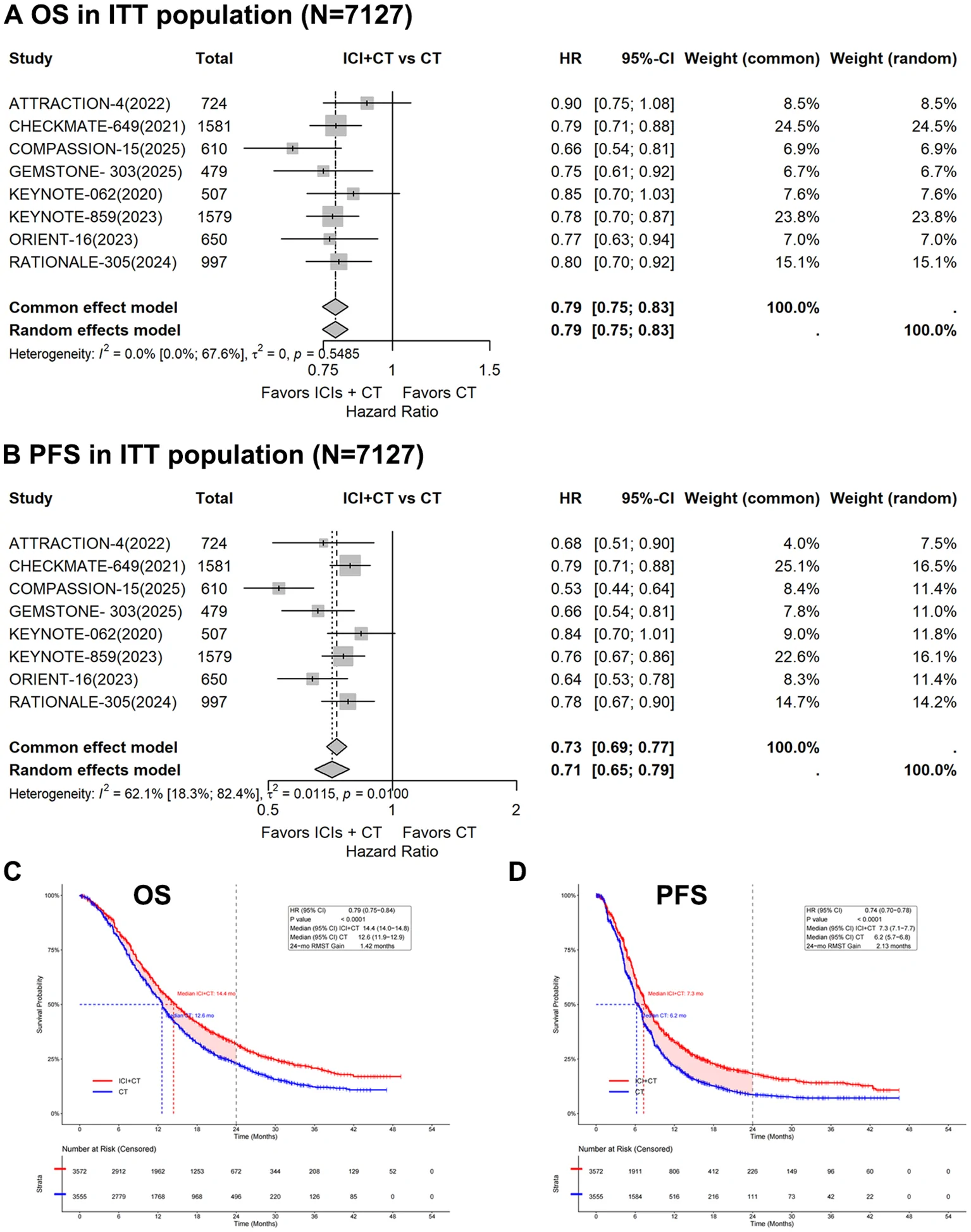
Forest plots and Kaplan-Meier estimates depicting pooled efficacy outcomes in the intention-to-treat (ITT) population: **(A)** overall survival (OS) and **(B)** progression-free survival (PFS) for immune checkpoint inhibitors plus chemotherapy (ICIs + CT) versus chemotherapy (CT) alone. **(C)** Kaplan-Meier curves for OS **(D)** Kaplan-Meier curves for PFS. The red line represents the immune checkpoint inhibitor plus chemotherapy (ICI+CT) group, and the blue line represents the chemotherapy (CT) group. Shaded areas indicate the 95% confidence intervals (CI). Vertical tick marks on the curves represent censored data.

In the pooled analysis, ICI plus chemotherapy significantly improved OS compared with chemotherapy alone (HR = 0.79, 95% CI 0.75–0.83; p < 0.001; I² = 0%, p_heterogeneity = 0.54) (**Figure 1A**). Similarly, a significant benefit was observed in PFS (HR = 0.71, 95% CI 0.65–0.79; p < 0.001; I²= 62.1%, p_heterogeneity = 0.01) (**Figure 1B**).

The reconstructed IPD yielded internally consistent patient-level time-to-event profiles across trials and enabled an integrated assessment of treatment effects. For OS, immunochemotherapy produced a median survival of 14.4 months compared with 12.6 months for chemotherapy alone. The corresponding 12-month OS rates were 57.16% and 50.81%, respectively. The hazard ratio for death was 0.79, reflecting a 21% relative reduction in mortality risk (**Figure 1C**). For PFS, the immunochemotherapy group achieved a median PFS of 7.3 months versus 6.2 months in the chemotherapy group, with 12-month PFS rates of 32.35% and 21.04%, respectively. The hazard ratio for progression or death was 0.74, indicating a 26% reduction in risk (**Figure 1D**). Collectively, these findings demonstrate a consistent survival benefit associated with immunochemotherapy following reconstruction and pooling of the original Kaplan–Meier data.

The temporal dynamics of survival benefit were further elucidated via RMST analysis. For overall survival, the absolute mean survival gain (ΔRMST) conferred by immunochemotherapy expanded from 0.38 months at the 12-month landmark to 1.42 months at the 24-month truncation horizon. A congruent pattern was observed for progression-free survival, with the ΔRMST incrementing from 0.88 to 2.13 months over the identical temporal span, corroborating the durable efficacy elicited by PD-1 blockade.

There was low-to-moderate heterogeneity among the trials (I² = 0% for OS; 62.1% for PFS). Funnel plots and Egger’s regression tests revealed no significant publication bias (Egger’s p = 0.83 for OS; p = 0.15 for PFS) (**Supplementary Figures 2A**, **S3A**). Galbraith (radial) plots showed that most studies clustered within 95% confidence limits (**Supplementary Figures 2B**, **S3B**). Sensitivity analyses confirmed the robustness of these results, with no single study unduly influencing the pooled estimates (**Supplementary Figures 2C**, **S3C**).

### Safety in ITT population

**Figure 2:**
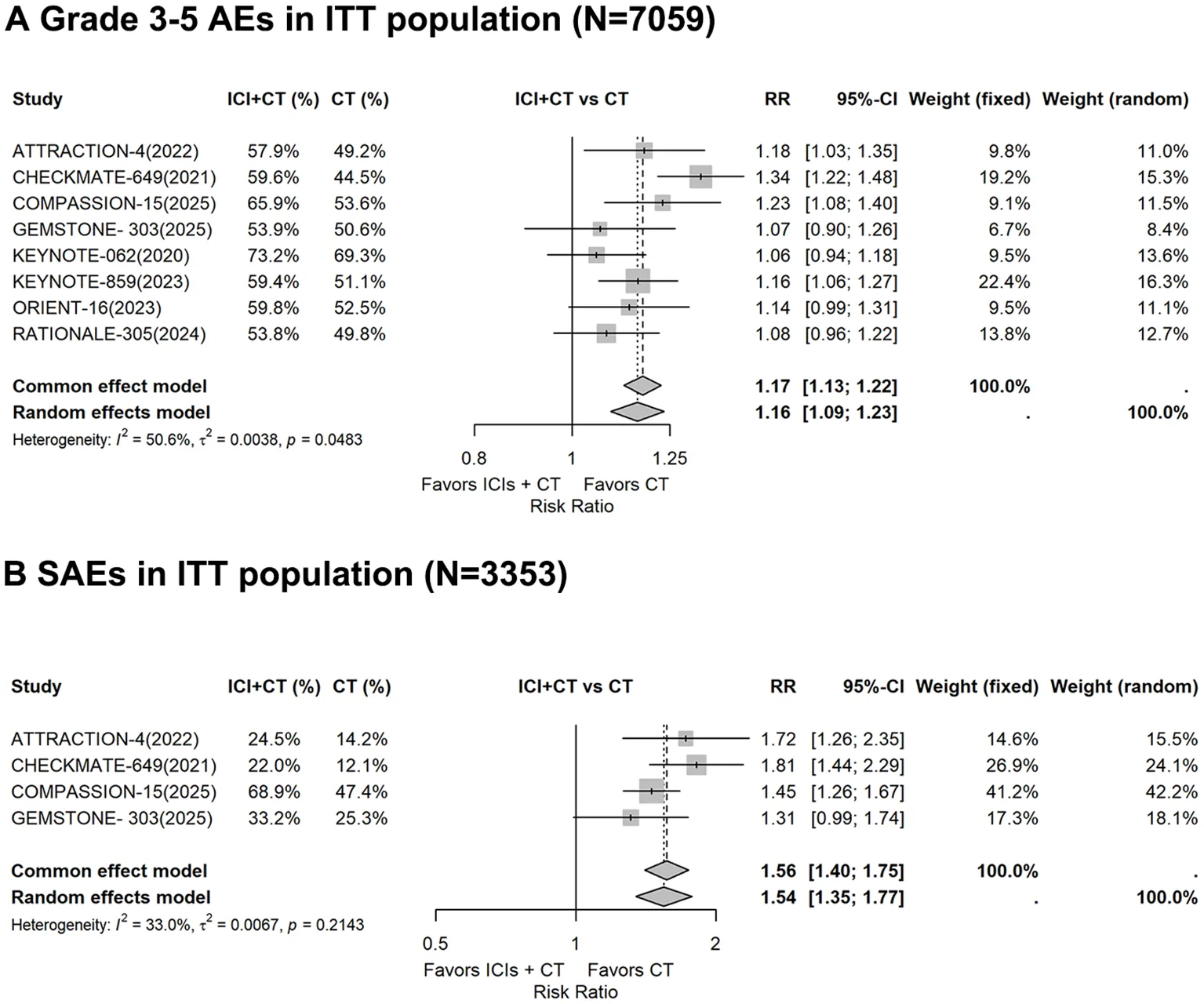
Forest plots illustrating safety outcomes in the intention-to-treat (ITT) population: **(A)** grade 3–5 adverse events (AEs) and **(B)** serious adverse events (SAEs) reported as relative risk (RR) for immune checkpoint inhibitors plus chemotherapy (ICIs + CT) versus chemotherapy (CT) alone.

The safety population included all the patients who received at least one dose of the assigned treatment. Combination therapy with ICI significantly increased the risk of grade ≥ 3 AEs (RR = 1.16, 95% CI 1.09–1.23; p < 0.001; I²= 50.6%, p_heterogeneity = 0.05) and SAEs (RR = 1.54, 95% CI 1.35–1.77; p < 0.001; I²= 33.0%, p_heterogeneity = 0.21) compared with chemotherapy alone (**Figures 2A, B**).

The most frequently reported high-grade AEs included neutropenia, anemia, and nausea, while immune-related AEs such as thyroid dysfunction, rash, and hepatitis were more common in the ICI group, but were generally manageable with corticosteroids.

No significant heterogeneity was detected across safety outcomes (I² = 50.6% for AEs; 33% for SAEs). Funnel, Galbraith, and sensitivity analyses (**Supplementary Figures 4, S5**) confirmed the stability of the pooled safety results, with no evidence of small-study bias.

### Subgroup analysis

Because certain studies lacked sufficient power to analyze clinically relevant subgroups, we conducted subgroup analyses based on patient demographics and tumor characteristics to better characterize the efficacy of ICIs combined with chemotherapy in specific subgroups and to inform personalized precision treatment.

### Patient-related subgroups

Across clinical subgroups stratified by demographic and performance characteristics, the OS and PFS benefits of ICI plus chemotherapy were largely consistent (**Supplementary Figures 6, 7**).

Patients aged <65 years and ≥65 years derived similar OS benefit (HR = 0.78 vs 0.79; interaction p = 0.77). Both males and females experienced comparable survival improvement (HR = 0.77 vs 0.81; interaction p = 0.39). Similarly, efficacy did not differ significantly by ECOG performance status (0 vs 1; HR = 0.80 vs 0.76; interaction p = 0.48) or by geographic region (Asia vs non-Asia; HR = 0.77 vs 0.81;interaction p = 0.44).

These results indicate that the clinical efficacy of ICIs combined with chemotherapy is broadly applicable across the major patient subpopulations.

### Tumor-related subgroups

Among tumor-related characteristics (**Supplementary Figure 8**), consistent OS and PFS benefits were observed regardless of primary tumor location in the gastric and GEJ (OS: HR = 0.77 vs 0.79;interaction p = 0.82; PFS: HR = 0.66 vs 0.67;interaction p = 0.87).

Moreover, patients with liver metastasis appeared to derive a slightly greater benefit (OS: HR = 0.72, 95% CI 0.64–0.80) than those without liver metastasis (OS: HR = 0.82, 95% CI 0.76–0.89), although the interaction did not reach statistical significance (p = 0.055).

### Analysis based on PD-L1 CPS expression

A gradient increase in the therapeutic benefit was observed with higher PD-L1 expression (**Figuress 3**, **4**). For OS, pooled HRs were 0.90 (CPS < 1), 0.76 (CPS ≥ 1), 0.74 (CPS ≥ 5), and 0.65 (CPS ≥ 10). The corresponding HRs for PFS were 0.87, 0.73, 0.65, and 0.63, respectively.

**Figure 3:**
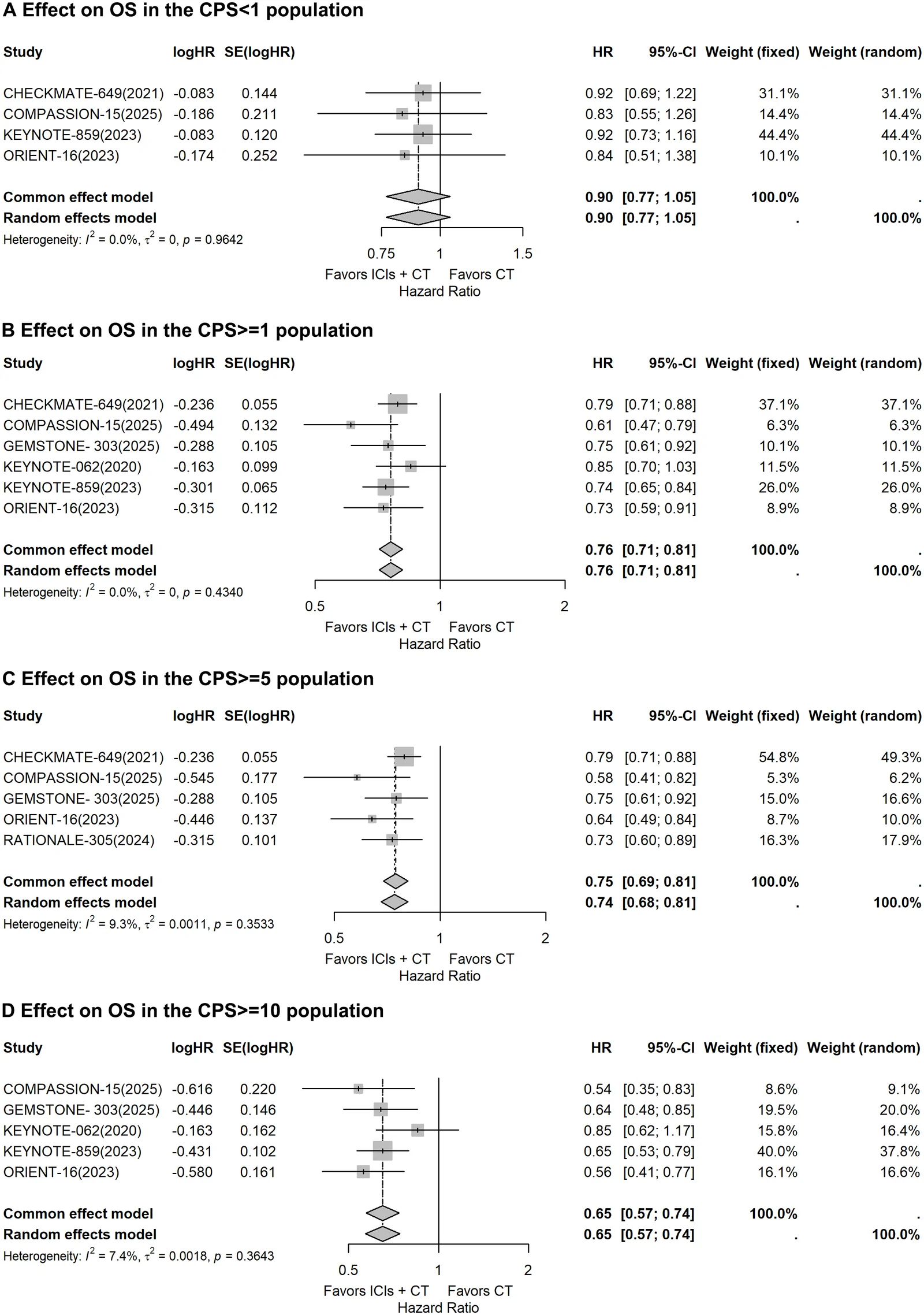
Forest plots displaying the results of overall survival (OS) stratified by the PD-L1 combined positive score (CPS) [**(A)**:<1; **(B)**≥1; **(C)**≥5; **(D)**≥10] in patients receiving immune checkpoint inhibitors plus chemotherapy (ICIs + CT) versus chemotherapy (CT) alone.

**Figure 4:**
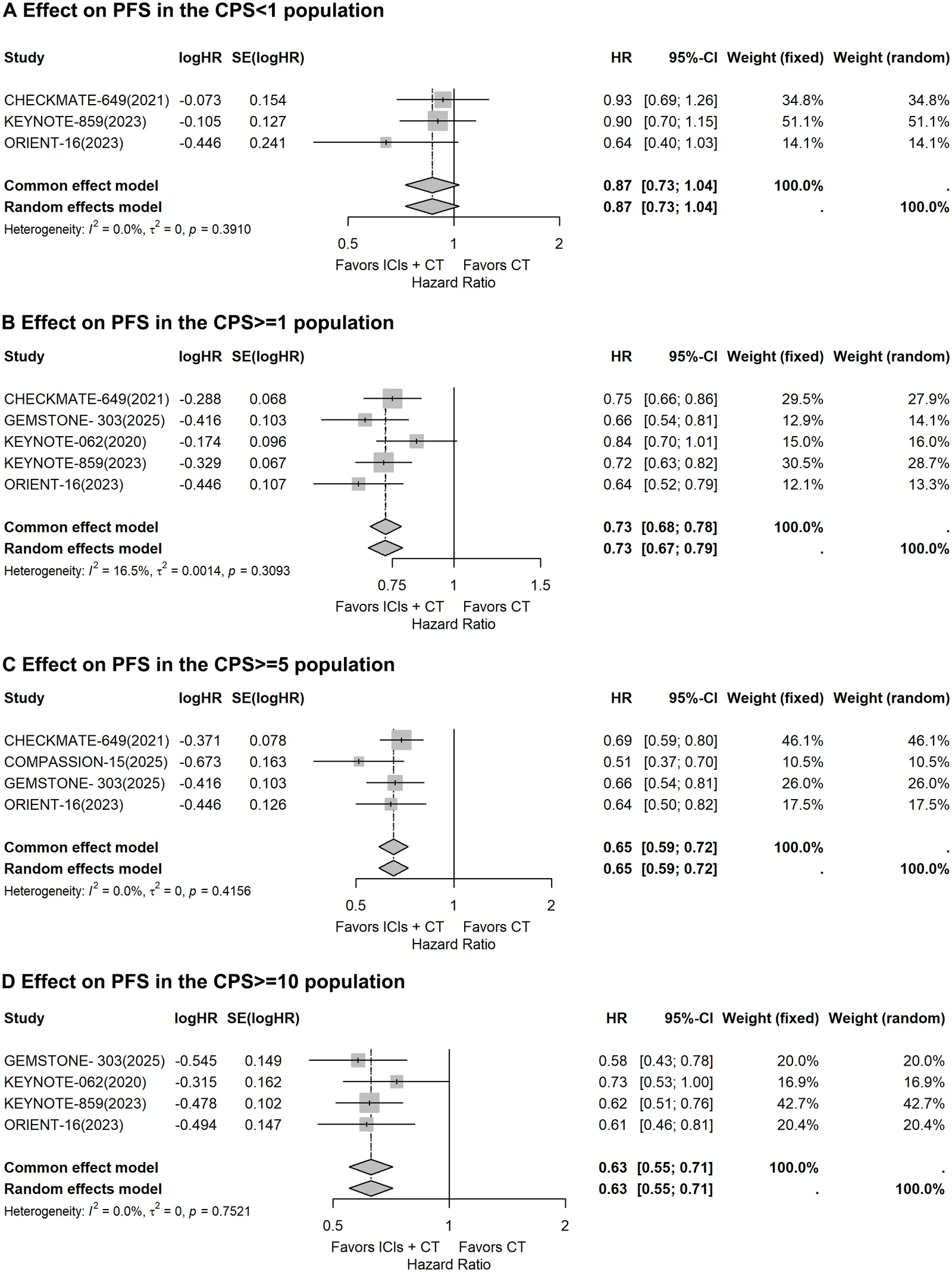
Forest plots displaying the results of progression-free survival (PFS) stratified by the PD-L1 combined positive score (CPS) [**(A)**:<1; **(B)**≥1; **(C)**≥5; **(D)**≥10] in patients receiving immune checkpoint inhibitors plus chemotherapy (ICIs + CT) versus chemotherapy (CT) alone.

**Figure 5:**
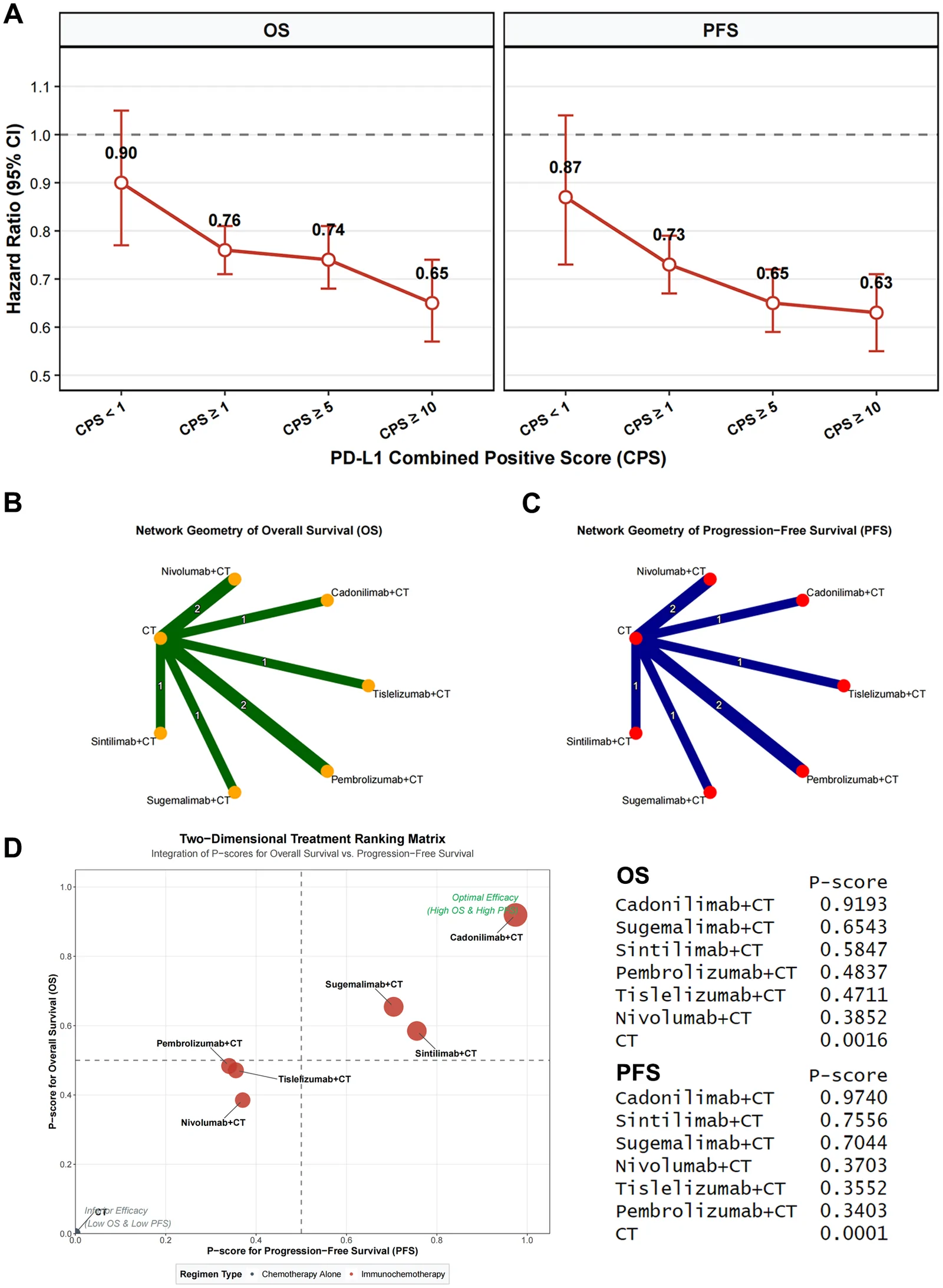
Efficacy Hierarchy and Drugs Select of First-Line Immunotherapy in Advanced Gastric Cancer: **(A)** Effect of the addition of immune checkpoint inhibitors (ICIs) to standard first-line chemotherapy (CT) on overall survival (OS) and progression-free survival (PFS) by increasing CPS cut-off. **(B, C)** Network geometry of the treatment comparisons for overall survival (OS) and progression-free survival (PFS). **(D)** Two-dimensional clustered scatter matrix illustrating the treatment ranking profiles.

To determine the clinically meaningful cutoff for PD-L1 expression, treatment effects were compared across CPS thresholds (**Figure 5A**). A significant improvement in OS and PFS was evident at CPS ≥ 1, with further deepening of the benefit at CPS ≥ 5 and ≥ 10, suggesting a dose–response relationship between PD-L1 expression and immunotherapy efficacy.

No clear survival advantage was observed in the CPS < 1 subgroup, supporting the use of CPS ≥ 1 as a minimal biomarker threshold for patient selection.

### Network meta-analysis and treatment ranking

The structural framework of the NMA exhibited a symmetric, star-shaped topology across both OS and PFS endpoints. Chemotherapy functioned as the central common comparator node. This configuration anchored the entire network, connecting six distinct immune checkpoint inhibitor regimens (Cadonilimab, Nivolumab, Pembrolizumab, Sintilimab, Sugemalimab, and Tislelizumab combined with CT). The exclusive reliance on this central node enabled robust indirect comparisons among the active interventions. The complete absence of closed triangular loops within the network geometry inherently precluded formal mathematical inconsistency between direct and indirect evidence (**Figures 5B, C**).

Within the frequentist network framework, treatment modalities were ranked hierarchically based on their P-scores. For both PFS and OS, the integration of immune checkpoint inhibitors uniformly outperformed chemotherapy alone. Notably, Cadonilimab plus chemotherapy demonstrated the highest likelihood of conferring optimal survival benefit, achieving peak P-scores for both OS (0.94) and PFS (0.98). Sugemalimab plus chemotherapy closely followed within this statistical hierarchy. The foundational anti-PD-1 agents, Nivolumab and Pembrolizumab, exhibited robust yet intermediate ranking probabilities across the broader immunochemotherapy cluster (**Figure 5D**).

## Discussion

This meta-analysis of eight phase III randomized controlled trials demonstrated that adding ICI to chemotherapy significantly improved OS and PFS in patients with advanced gastric and GEJ adenocarcinoma. The pooled hazard ratios (HR = 0.79, OS and HR = 0.71, PFS) confirmed a clinically meaningful benefit. Given that the delayed therapeutic onset of immune checkpoint inhibitors frequently violates traditional proportional hazards assumptions, RMST provides a robust, assumption-free metric to quantify absolute patient benefit. Within our 24-month observation horizon, the immunochemotherapy cohort achieved a clinically meaningful absolute overall survival extension of 1.42 months relative to chemotherapy alone. This expanding survival advantage over time fundamentally corroborates the durable and profound immune-mediated efficacy characteristic of frontline PD-1 blockade in advanced gastric cancer.

The consistency of benefits across trials underscores the robustness of PD-1 blockade in this malignancy, which historically showed modest chemosensitivity and poor survival outcomes. These findings extend the results of pivotal trials such as CheckMate-649, KEYNOTE-062, KEYNOTE-859, and ORIENT-16, where nivolumab, pembrolizumab, or sintilimab combined with chemotherapy improved survival compared with chemotherapy alone (5, 7, 9, 17). The biological rationale involves synergy between chemotherapy-induced antigen release and PD-1 blockade–mediated T-cell reactivation, leading to enhanced immune-mediated cytotoxicity (20, 21).

The landscape of first-line therapy for advanced gastric cancer has become increasingly complex with the emergence of multiple PD-1 and PD-L1 inhibitors. Our network meta-analysis addressed the lack of head-to-head trials by establishing a competitive hierarchy through P-score ranking. Based on probabilistic rankings, Cadonilimab plus chemotherapy emerged as the regimen with the highest likelihood of yielding optimal outcomes for both OS and PFS. The superior ranking of Cadonilimab, a bispecific PD-1/CTLA-4 antibody, underscores the potential synergistic effect of dual-checkpoint blockade in overcoming primary resistance within the tumor microenvironment. While conventional anti-PD-1 agents such as Nivolumab and Pembrolizumab remain standard-of-care, the P-score distribution suggests that novel molecular architectures or multi-target inhibitors may elicit deeper and more sustained pathological responses.

A key finding of this analysis was the gradient of efficacy with increasing CPS. OS hazard ratios decreased from 0.90 in CPS <1 to 0.76, 0.74, and 0.65 in CPS ≥1, ≥5, and ≥10, respectively. Conceptually, this progressive trend strongly supports the paradigm of viewing PD-L1 CPS not merely as a rigid binary threshold, but rather as a continuous biological predictor of ICI efficacy in advanced gastric cancer. Nonetheless, a subset of tumors with low or negative PD-L1 expression may still derive clinical benefits from PD-1 blockade (22), suggesting the existence of interferon-γ (IFN-γ)–independent immune activation pathways. These mechanisms may involve alternative stimulatory signals such as type I interferons, tumor antigen cross-presentation, or metabolic and epigenetic reprogramming of tumor-infiltrating lymphocytes (22–24). Recognizing these IFN-γ–independent responses aligns with emerging concepts in checkpoint biology and highlights that PD-L1 expression, while useful, does not fully capture the complexity of immune responsiveness in gastric and GEJ adenocarcinoma. PD-L1 expression correlates with interferon-γ signaling, tumor-infiltrating lymphocyte density, and cytolytic gene activity (25, 26). However, the interpretation of PD-L1 subgroup analyses requires considerable caution due to inherent methodological heterogeneity across the synthesized trials. Distinct immune checkpoint inhibitors utilize tightly paired companion diagnostic assays (e.g., 22C3 for Pembrolizumab, 28–8 for Nivolumab) deployed on divergent immunohistochemical platforms. Furthermore, the evaluation algorithms vary structurally, encompassing the CPS, tumor proportion score (TPS), and tumor area positivity (TAP) (27). These diverse methodologies evaluate biologically distinct cellular compartments—incorporating varying proportions of immune infiltrates—and frequently exhibit suboptimal analytical interchangeability (28). Consequently, amalgamating these disparate diagnostic metrics into a unified biomarker cohort introduces an unquantifiable confounding factor. Such intrinsic assay discordance may obscure true predictive thresholds and highlights the critical urgency for global PD-L1 harmonization initiatives in gastroesophageal oncology.

Beyond PD-L1, multiple tumor-intrinsic and immune-related factors influence checkpoint responses, including microsatellite instability, Epstein–Barr virus status, tumor mutational burden, and T-cell–inflamed gene expression profiles (29, 30). Integrating these features into composite models could enhance the predictive accuracy, especially for CPS-low tumors with heterogeneous immune landscapes.

Interestingly, patients with liver metastases may exhibit survival benefit from ICI-based therapy. This observation contrasts with earlier reports suggesting that the liver is an immunosuppressive “sink.” The hepatic microenvironment typically contains tolerogenic antigen-presenting cells and cytokines, such as TGF-β and IL-10, which dampen systemic antitumor immunity (31, 32). Cytotoxic chemotherapy may partially reverse this immunological suppression by inducing immunogenic cell death, releasing tumor antigens, and reducing myeloid-derived suppressor cells. These mechanisms may transiently sensitize the hepatic metastases to immune modulation. Clinically, this trend reinforces that patients with liver metastases should not be excluded from immunotherapy and highlights the need for translational studies examining hepatic immune remodeling.

Regarding safety, combination therapy predictably increased the grade ≥3 adverse events (RR = 1.16) and serious adverse events (RR = 1.54). The main contributors were chemotherapy-related cytopenia and gastrointestinal toxicity, as well as immune-related thyroid, hepatic, and cutaneous events. Most immune-mediated toxicities are manageable with corticosteroids and rarely lead to fatal outcomes. These findings are consistent with prior pooled analyses confirming that PD-1 inhibitor–chemotherapy combinations maintain an acceptable toxicity profile. In clinical decision-making, incremental toxicity must be weighed against survival gain, but the absolute benefit in OS and PFS clearly outweighs manageable risks (33, 34). Early recognition and multidisciplinary management of immune-related events are crucial for optimizing outcomes.

This meta-analysis has several limitations. First, heterogeneity among the trials must be acknowledged. Different PD-L1 evaluation systems (CPS, TPS, and TAP) and cut-offs were used, which may account for residual statistical heterogeneity and hinder direct cross-study comparisons. Second, the subgroup meta-analysis in this study was based on aggregate data rather than IPD. Without IPD, adjusting for covariates or exploring time-dependent effects is impossible, limiting the precision of the subgroup analyses. Moreover, only eight phase III trials were available, all of which were industry-sponsored, raising the possibility of publication or reporting bias despite the symmetrical funnel plots. Independent replication and open data sharing are important to validate these findings. Future IPD meta-analyses could refine biomarker thresholds and clarify treatment effects across patient-level covariates (35). Third, several trials (ATTRACTION-4, ORIENT-16, and GEMSTONE-303) primarily enrolled Asian populations, potentially limiting generalizability to Western settings where biological and clinical characteristics differ. Finally, while CheckMate-649 is an open-label trial and theoretically at risk of performance bias, key endpoints were objectively assessed. Funnel plot symmetry and sensitivity analyses showed no evidence of publication bias, thereby supporting the robustness of the pooled results.

In summary, ICI plus chemotherapy provides substantial and consistent survival benefits across the clinical and biological subgroups of advanced gastric and GEJ adenocarcinoma. The efficacy gradient by CPS reinforces PD-L1 expression as a clinically actionable biomarker, whereas the trend toward greater benefit in patients with liver metastases suggests a possible reversal of hepatic immune resistance under combination therapy. Although the toxicity burden is high, it remains manageable within standard supportive frameworks. Future research should prioritize PD-L1 assay harmonization, integration of multi-omic biomarkers, IPD meta-analyses, and prospective optimization of therapy duration and sequencing. Collectively, these efforts will advance precision immunotherapy and further improve the outcomes of patients with this aggressive disease.

## Clinical implications

The findings of this meta-analysis are directly relevant to the current clinical practice guidelines. Both the National Comprehensive Cancer Network (NCCN, 2025) and the European Society for Medical Oncology (ESMO, 2024) now recommend PD-1 inhibitor plus chemotherapy as the preferred first-line regimen for patients with HER2-negative, advanced gastric, or GEJ adenocarcinoma whose tumors express PD-L1 with a CPS ≥1. Our results reinforce these recommendations by demonstrating that the magnitude of the benefit from immune checkpoint inhibitor–based combination therapy increases progressively with higher CPS thresholds (≥1, ≥5, and ≥10). This supports routine PD-L1 testing using validated CPS assays as essential biomarkers for treatment selection and patient counseling. Furthermore, the consistent efficacy observed across age, sex, ECOG PS, and geographic subgroups underscores the broad applicability of PD-1 blockade, while the manageable toxicity profile supports its integration into standard practice. Future updates to the NCCN and ESMO guidelines should continue to refine biomarker-driven patient selection, incorporating PD-L1 expression alongside emerging genomic and immune signatures, to optimize individualized therapy for advanced gastric and GEJ adenocarcinoma.

## Data Availability

The raw data supporting the conclusions of this article will be made available by the authors, without undue reservation.
