# Supplementary material for: Efficacy hierarchy and absolute survival benefit of first-line immunotherapy in advanced gastric cancer: a meta-analysis and pooled survival analysis
Source: Front Immunol. 2026 Aug 17;17:1866848. doi: 10.3389/fimmu.2026.1866848 (PMC13526996; doi:10.3389/fimmu.2026.1866848)
Supplement: Supplementary file 1 [file SupplementaryFile1.docx]

**FigureS1**

Flow diagram for the selection of the studies included in the meta-analysis

**Figure S2**

Funnel plots (A), Galbraith plots (B) and Sensitivity analyses (C) for included studies examined by leaving-one-out approach for overall survival (OS).

**Figure S3**

Funnel plots (A), Galbraith plots (B) and Sensitivity analyses (C) for included studies examined by leaving-one-out approach for progression-free survival (PFS).

**Figure S4**

Funnel plots (A), Galbraith plots (B) and Sensitivity analyses (C) for included studies examined by leaving-one-out approach for grade 3–5 adverse events (AEs).

**Figure S5**

Funnel plots (A), Galbraith plots (B) and Sensitivity analyses (C) for included studies examined by leaving-one-out approach for serious adverse events (SAEs).

**FigureS6**

Forest plots displaying the results of overall survival (OS) and progression-free survival (PFS) stratified by (A, B) age and (C, D) sex in patients receiving immune checkpoint inhibitors plus chemotherapy (ICIs + CT) versus chemotherapy (CT) alone.

**FigureS7**

Forest plots displaying the results of overall survival (OS) and progression-free survival (PFS) stratified by (A, B) Eastern Cooperative Oncology Group (ECOG) performance status and (C, D) residential area.

**FigureS8**

Forest plots displaying the results of overall survival (OS) and progression-free survival (PFS) stratified by (A, B) tumor site and (C, D) liver metastasis.
